# Preparation and *In Vitro*/*In Vivo* Evaluation of Vinpocetine Elementary Osmotic Pump System

**DOI:** 10.1155/2011/385469

**Published:** 2011-04-10

**Authors:** Meiying Ning, Yue Zhou, Guojun Chen, Xingguo Mei

**Affiliations:** ^1^Novel Drug Delivery and Biomaterial Research Center, National Research Institute for Family Planning, Haidian District Dahuisi Road 12, Beijing 100081, China; ^2^Beijing Institute of Pharmacology and Toxicology, Taiping Road 27. Haidian District, Beijing 100850, China

## Abstract

Preparation and *in vitro* and *in vivo* evaluation of vinpocetine (VIN) elementary osmotic pump (EOP) formulations were investigated. A method for the preparation of VIN elementary osmotic pump tablet was obtained by adding organic acid additives to increase VIN solubility. VIN was used as the active pharmaceutical ingredient, lactose and mannitol as osmotic agent. Citric acid was used as increasing API solubility and without resulting in the API degradation. It is found that the VIN release rate was increasing with the citric acid amount at a constant range. Cellulose acetate 398-3 was employed as semipermeable membrane containing polyethylene glycol 6000 and diethyl-o-phthalate as pore-forming agent and plasticizer for controlling membrane permeability. In addition, a clear difference between the pharmacokinetic patterns of VIN immediate release and VIN elementary osmotic pump formulations was revealed. The area under the plasma concentration-time curve after oral administration of elementary osmotic pump formulations was equivalent to VIN immediate release formulation. Furthermore, significant differences found for mean residence time, elimination half-life, and elimination rate constant values corroborated prolonged release of VIN from elementary osmotic pump formulations. These results suggest that the VIN osmotic pump controlled release tablets have marked controlled release characters and the VIN osmotic pump controlled release tablets and the normal tablets were bioequivalent.

## 1. Introduction

Vinpocetine (VIN) is a vasoactive vinca alkaloid and a synthetic derivative of apovincamine which has been used in clinical practice in Europe for 30 years for the treatment of disorders arising from cerebrovascular and cerebral degenerative diseases [1, 2]. VIN is thought to increase the cerebral blood flow in the ischemic areas of patients with cerebrovascular disease, decrease platelet aggregability in patients with transient ischemic attack or stroke, increase red blood cell deformability in stroke patients, and have neuroprotective abilities and a protective effect against brain ischemia [3, 4].

VIN is mainly used as immediate oral dosage forms containing 5 mg of the active ingredient, with a daily dosage regimen that can vary between 5 mg × 3/day to 20 mg × 3/day [5]. Conventional preparation will lead to large fluctuation in drug plasma concentration and side effect on human body. Constant plasma level can offer a therapeutic advantage for many drugs in terms of both efficacy and tolerance of the treatment [6]. Once-daily controlled release preparation is often desirable. 

Research about sustained-release formulations of VIN had been reported. Ribeiro et al. [7, 8] reported that Vin-cyclodextrin-tartaric acid multicomponent complexation was prepared and an optimal formulation was then designed by the combination of these complexes into HPMC-based hydrophilic tablet dosage forms. Results supported the use of HPMC matrices to provide a useful tool in retarding the release of VIN and that dissolution characteristics of the drug may be modulated by multicomponent complexation in above delivery systems. Nie et al. [9] had successfully prepared HPMC sustained-release matrix tablets containing citric acid. Cao et al. [10] reported the push-pull osmotic pump tablet preparation of VIN and* in vivo*/*in vitro* evaluation.

The osmotic pump tablet that holds a prominent place among controlled release systems has many advantages, such as reducing the risk of adverse reactions, improving compliance of patients, and exhibiting comparable *in vitro*/*in vivo* drug release. 

**Scheme 1:**
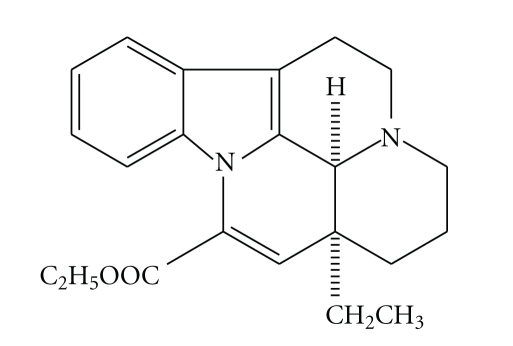
The chemical structure of VIN.

The first osmotic pump for delivery of active ingredients was invented by Rose and Nelson in the 1950s [11]. The first commercial osmotic device was introduced by Theeuwes known as elementary osmotic pump (EOP) in the 1970s [12]. The EOP was a core tablet coated by semipermeable membrane with a micro-orifice drilled on the surface. The EOP was simpler than push-pull osmotic pump reported by Cao et al. [10] in preparation and could deliver water-soluble drugs at an approximately constant rate up to 12 h. However, VIN is a poorly water-soluble base type drug (Scheme 1). So, it is critical to improve drug solubilities. It was reported that a method for the preparation of water-soluble drug was obtained by adding acid additives to increase dissolution and improve the release profile [13, 14]. In the present paper, preparation with citric acid in core tablets and *in vitro/in vivo *evaluation were investigated. 

## 2. Materials and Methods

**Materials**

VIN (Changchun Pharmaceutical Co., Ltd., China) was API. Citric acid (Beijing Fengli Jingqiu Ltd., China) was used as increasing dissolution. Lactose (Beijing Fengli Jingqiu Ltd., China) and mannitol (Beijing Fengli Jingqiu Ltd., China) were used as osmotic agent. PVP K30 (Beijing Fengli Jingqiu Ltd., China), HPMC K4M (Beijing Fengli Jingqiu Ltd., China), and Magnesium Steric (Beijing Fengli Jingqiu Ltd., China) were used as binder, retarder, and lubricate, respectively. Cellulose acetate 398-3 (Estman Co., Ltd., USA) was employed as semipermeable membrane containing polyethylene glycol 6000 (PEG-6000, Beijing Fengli Jingqiu Ltd., China) as plasticizer, Diethyl-o-phthalate (Beijing Jingqiu Ltd., China) as pores-forming agent, and acetone (Beijing Chemical Agent Ltd., China) as solvent for controlling membrane permeability. Other chemicals used were of analytical grade.

**Preparation of Core Tablets**

VIN powder was mixed with lactose, mannitol, HPMC K4M, and PVP K30 manually using 70% ethanol solution as binder, and then the mixture was passed through an 850 ± 29 *μ*m sieve to generate granules and dried at 45°C for 4 h. Then the above-mentioned mixture was sifted by a 1375-*μ*m sieve. The resultant granules were compressed into core tablet using the PXD single-punch tableting machine (Beijing Hangtian Changfeng Pharmaceutical Device Co., Ltd., China). The weight of each core tablet was maintained within the range of (180 ± 5%) mg. The hardness of the core tablets was kept within (60~120) *N* (Hardness tester, Shanghai Huanghai Drug Inspection Instrument Co., Ltd., China). The relative humidity of surrounding environment should be kept below 50%.

**Coating and Drying**

CA in 95% acetone (%, w/v) containing plasticizer (PEG-6000) and diethyl-o-phthalate was used as coating solution. The coating was carried out in a pan coater (BY300·fa, Jiangsu Taizhou Drug Development Instrument Co., Ltd., China). The temperature of coating pan was 30~40°C; pan-rotating rate was 20 rpm. The coated tablets were dried to remove the residual solvent acetone and water at 40°C for 24 h, and laser machines (Jiangsu Meilin Co., Ltd., China) drilled an orifice on the membrane surface.

**UV Analysis of VIN**

The detection wavelength was 268 nm. Linear correlation was obtained between peak area versus concentration of VIN in the range of 2.118~31.77 *μ*g/mL. Each measurement represented the average of three replicates. The regression equation was found to be *A* = 0.0317*C* + 0.0046, *r* = 1.000, where *A* is the peak area and *C* is the concentration of VIN in 2.118~31.77 *μ*g/mL.

***In Vitro* Release Test**

*In vitro* drug release test was conducted in a dissolution apparatus (D-800LS, Precise Apparatus of Tianjin University Co., Ltd, China) using the Basket method according to Ch.P (Chinese Pharmacopoeia edition 2005). The temperature of the release medium was maintained at 37 ± 0.5°C and the rotation speed of the basket was adjusted to 100 rpm. Samples of 10 mL were withdrawn at 2.0, 4.0, 6.0, 8.0, 12.0, 14.0, and 24.0 h and replaced 10.0 mL fresh release media to each vessel. The solution was immediately filtered through a 0.45 *μ*m membrane filter, suitably diluted and determined at 268 nm using the Cintra 10e spectrophotometer. All experiments were performed in triplicate.

***In Vivo* Studies Pharmacokinetic Studies in Dogs**

Regarding all animal studies, the research adhered to the “Principles of Laboratory Animal Care”. For pharmacokinetic studies, a group of three male beagle dogs was used and the formulations were administered in a crossover fashion. The VIN formulations were administered to fasted dogs and immediately followed by an oral gavage of 50 mL water. As a control, an immediate-release tablet formulation (Zhejiang Tailison Pharmaceutical Co. Ltd.) was administered.

After dosing, dogs were returned to metabolism cages equipped with automated water supplies. They were fed their normal diet 8 h after dosing on the day of the study. The dogs remained in the metabolism cages until all of the tablets had been recovered. Whole blood samples (2.0 mL) were taken from the jugular vein of each dog using 5-mL disposable syringes before dosing and at 0.5, 1, 1.5, 2, 3, 4, 6, 8, 10, 14, 24, 30, 36, and 48 h after dosing for immediate release tablets/at 1, 2, 3, 4, 6, 8, 10, 14, 24, 30, 36, 48, and 60 h after dosing for controlled release tablets. The samples were immediately transferred to heparinized (sodium heparin) Vacutainersk. Samples were spun in a 5°C centrifuge at 3000 rpm for 5 min. The plasma samples were poured into 2-mL plastic tubes. Samples were frozen and stored in a freezer until they were analyzed for VIN, usually within a period of 1 month. HPLC-MS-MS (Aglient 1200HPLC + Agilent 6410 Triple Quad LC/MS (ESI)) method was to be adopted to analyse *in vivo* samples.

The plasma-concentration-versus-time profiles were analyzed using standard pharmacokinetic analysis techniques. Peak plasma concentrations (*C*_max_) were determined by inspection of the data. The first occurrence of *C*_max_ was defined as the *T*_max_. Areas under the plasma-concentration-time curves (AUC) were calculated by the linear trapezoidal method using 3P97 Professional (Chinese pharmacologicalassociation software). Pharmacokinetic parameters were dose-normalized for ease of comparison with the immediate-release control. Relative bioavailability (RBA) was defined as the dose-corrected AUC_0-∞_ of the osmotic pump controlled release formulation divided by the dose-corrected AUC_0-∞_ of the immediate-release tablet. 

## 3. Results and Discussion

### 3.1. Influence of Citric Acid on Drug Release Profile

**Figure 1:**
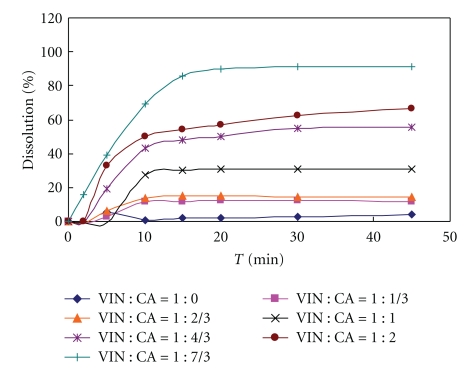
Influence of molar ratio of citric acid and vinpocetine on drug dissolution profile (core tablets in water dissolution media); VIN: vinpocetine; CA: citric acid.

In order to study the influence of citric acid amount on drug dissolution, core tablets with various molar ratios of VIN and citric acid formulations in Table 1 were prepared and were investigated. Dissolution profile in water.  Figure 1 showed that the VIN dissolution was increasing as the citric acid amount increased, which was in accordance with the report [9]. When the molar ratio of VIN and citric acid was 1 : 7/3, the cumulative dissolution was above 90%. It could be concluded that the ratio (1 : 7/3) should be much more suited for the solubilization of VIN. 

**Figure 2:**
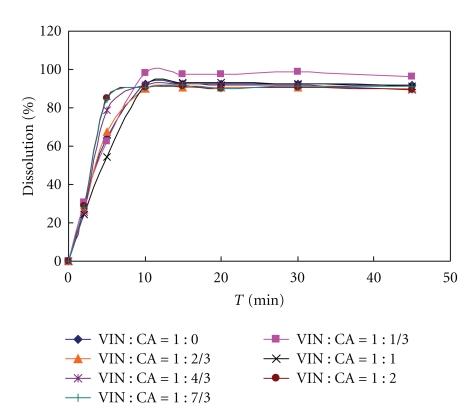
Formulation with different molar ratio citric acid and vinpocetine dissolution profile (core tablets in 0.1 N HCL dissolution media); VIN: vinpocetine; CA: citric acid.

**Figure 3:**
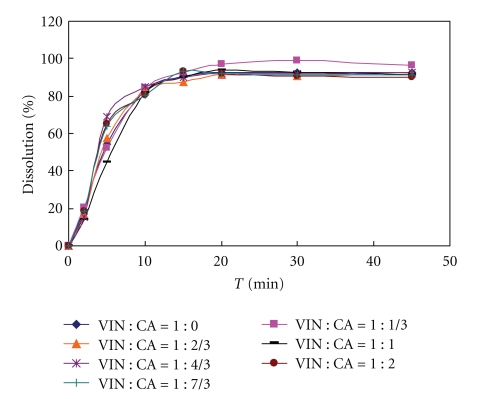
Formulation with different molar ratio citric acid and vinpocetine dissolution profile (core tablets in 0.5% SDS dissolution media); VIN: vinpocetine; CA: citric acid.

Then, tablets with various ratios of VIN and citric acid formulations were prepared coated with the same coating formulation (Table 3) to study the influence of citric acid amount on drug release in two different release media (0.1 N HCL, 0.5% SDS solution). The VIN could be dissolved in those two release media (see Figures 2 and 3) in order to investigate the function of citric acid. And another aim was to study whether H^+^ existing in release media could be penetrating through the semipenetrating membrane to influence the drug release, whose result should become a basis for selecting optimal release media.

**Figure 4:**
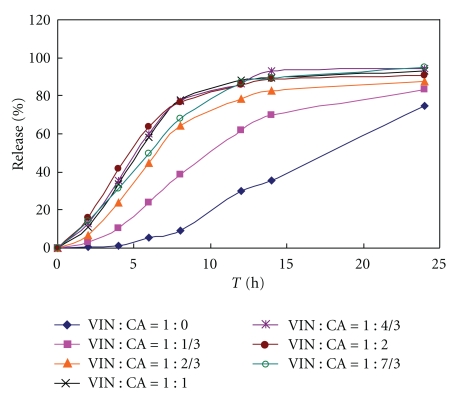
Influence of molar ratio of citric acid and vinpocetine on drug release profile (coating tablets in 0.1 N HCL); VIN: vinpocetine; CA: citric acid.

**Figure 5:**
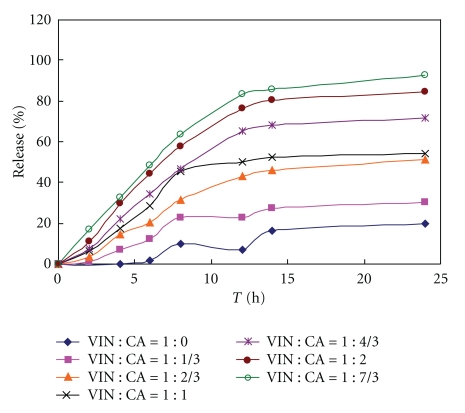
Influence of molar ratio of citric acid and vinpocetine on drug release profile (coating tablets in 0.5% SDS); VIN: vinpocetine; CA: citric acid.

Figures 4 and 5 showed that the amount of citric acid had a marked influence on VIN release. The tablet release rate and cumulative release at 12 h increased as the amount of citric acid increased. Citric acid played a critical role in promoting dissolving agent. It increased the dissolution of VIN and subsequently prevented precipitation of VIN. 

Figures 4 and 5 also showed that drug release of each time point in 0.1 N HCL media was higher than in 0.5% SDS solution, especially after 8 h. On the other hand, the result showed that in 0.1 N HCL media the cumulative drug release percents of formulations whose ratios of VIN and citric acid were higher than 1 : 1 had no difference and all above 90% after 24 h; however, in 0.5% SDS the formulations was 1 : 7/3. The results suggested that certain of H^+^ in release media could penetrate through the semipenetrating membrane which resulted in increasing the drug dissolubility. Thereof, only the VIN of core tablet should meet certain dissolubility, could different media be applied to test different formulations.

Table 2 showed that cumulative release of coating tablets in 0.5% SDS were higher than core tablets in water, which suggested that certain amount of drug could be pumped from the drug release pore by the osmotic pressure power. The results reproved the mechanism of osmotic pump tablet.

**Figure 6:**
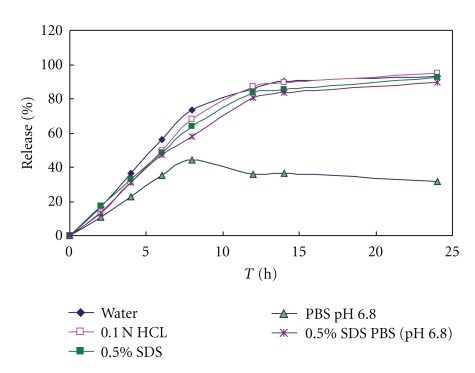
Release profile of optimal osmotic pump tablets formulation in different release media: water, 0.1 N HCL, 0.5% SDS, PBS pH 6.8, 0.5% SDS PBS (pH 6.8).

In the following, we selected different release media-water, 0.1 N HCL, 0.5% SDS solution, 0.5% SDS PBS (pH 6.8), and PBS (pH 6.8) to test the release profile. The release profiles (Figure 6) in-water, 0.1 N HCL, 0.5% SDS solution, 0.5% SDS PBS (pH 6.8) had no differences; but in PBS (pH 6.8) was slower, especially cumulative release decreasing after 8 h which suggested the VIN-H^ +^ had changed into free base in pH 6.8 condition. So, it could be deduced that the citric acid in formulation had a little effect on the structure and properties of VIN and absorption in the intestine.

**Figure 7:**
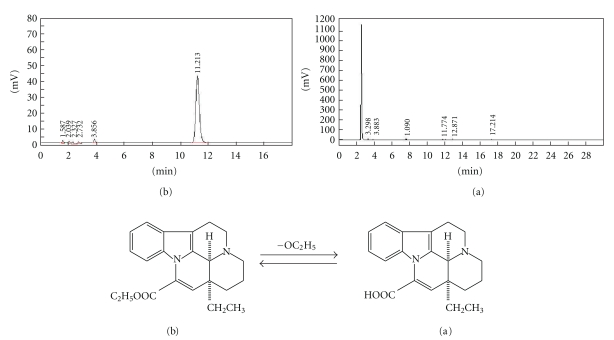
Chromatograph of de-acetyl-vinpocetine (a) and vinpocetine (b).

In order to validate thoroughly whether the structure of VIN changed, release solution after 24 h of formulation and de-acetyl-VIN had been detected by HPLC (Figures 7(a) and 7(b)). It was found that the chromatograph peak of de-acetyl-VIN did not appear in the release solution of formulation after 24 h, which indicated that the citric acid had no effect on the structure of VIN. It was suggested that the citric acid could have had a relation with changing the drug microenvironment pH to increase the drug dissolubility and release percent, which was different from the viewpoint [10].

The optimal VIN EOPT was found to be able to deliver VIN at a rate of approximately zero order up to 12 h in water, cumulative release at 12 h is above 80%. From the drug release mechanism of the EOPT, the application of EOPT designed in the current study was dependent on the drug solubility with increasing the organic acid. In conclusion, VIN EOPT designed in the present study was a good controlled delivery system for water-insoluble base type drugs. During the developing VIN elementary osmotic pump tablet, it is found that H^+^ in release media could be penetrating through the semipermeable membrane and could be influencing the pH-dependent insoluble drug release. In the following, in vivo evaluation was investigated. 

### 3.2. Pharmacokinetic Studies

**Figure 8:**
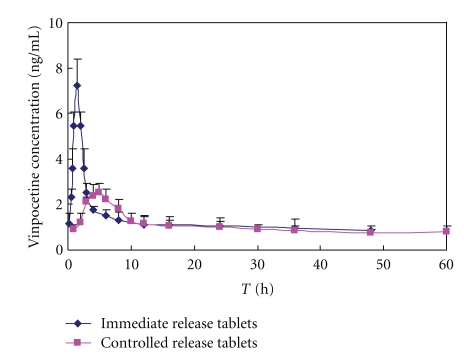
The mean vinpocetine plasma concentration-time curves of vinpocetine osmotic pump controlled-release tablets and immediate release tablets after oral administration in dogs (*n* = 6).

**Figure 9:**
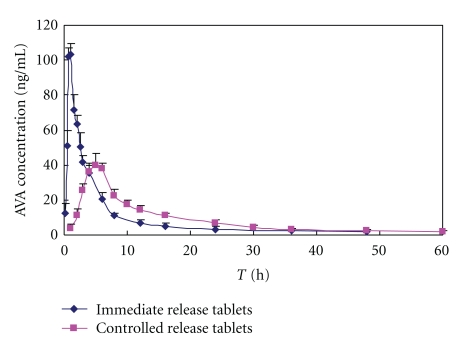
The mean vinpocetine metabolite apovincaminic acid (AVA) plasma concentration-time curves of vinpocetine osmotic pump controlled-release tablets and immediate release tablets after oral administration in dogs (*n* = 6, VIN: Vinpocetine).

The pharmacokinetics of VIN and its main metabolite, apovincaminic acid (AVA), were studied in dogs. The mean dose-normalized plasma VIN and apovincaminic acid (AVA) concentration-versus-time profiles following administration of the EOP formulations in beagle dogs are shown in (Figures 8 and 9). The pharmacokinetic parameters for VIN and apovincaminic acid (AVA) are summarized in Tables 4 and 5.

The pharmacokinetic parameters of osmotic pump tablets and immediate release tablets for VIN and its main metabolite, apovincaminic acid (AVA), were *T*_max_ (1.54 ± 0.64 h) and (4.83 ± 2.32 h), (1.67 ± 0.31 h) and (4.83 ± 1.02 h), *C*_max_ (10.21 ± 1.55 ng/mL) and (3.62 ± 1.34 ng/mL), and (149.28 ± 31.22 ng/mL) and (377.19 ± 39.89 ng/mL); the relative bioavailability of the controlled release tablets was 117.44%, 83.23%. Data in Figures 8 and 9 show similar data published about VIN using orally administered [11] C-labelled VIN and positron emission tomography in humans. Gulyás et al. [15] reported the *T*_max_ of immediate release tablets is about 2 h in humans by the above method which could be only in the initial distribution phase following oral administration due to the fact that the half-life of [11] C-labelled (20 min). 

A clear difference between the pharmacokinetic patterns of VIN immediate release and VIN elementary osmotic pump formulations was revealed. The area under the plasma concentration-time curve after oral administration of elementary osmotic pump formulations was equivalent to VIN immediate release formulation. Furthermore, significant differences (*P* < .05, *t*-test) found for mean residence time, *T*_max_ and *C*_max_ (Tables 4 and 5), corroborated prolonged release of VIN from elementary osmotic pump formulations. These results suggest that the VIN (water-insoluble base type drug) osmotic pump controlled release tablets with citric acid have marked controlled release characters and the VIN osmotic pump controlled release tablets and the normal tablets were bioequivalent. 

## Figures and Tables

**Table 1:** Composition for core tablets with various molar ratios of vinpocetine and citric acid (unit : mg).

Vin : citric acid	1 : 0	1 : 1/3	1 : 2/3	1 : 1	1 : 4/3	1 : 6/3	1 : 7/3
Vinpocetine	15.0	15.0	15.0	15.0	15.0	15.0	15.0
Citric acid	0	2.74	5.48	8.22	10.96	16.44	19.2
Lactose	70.17	69.25	68.34	67.43	66.51	64.69	63.77
Mannitol	140.33	138.5	136.68	134.85	133.02	129.37	127.53
HPMCK4M	7.5	7.5	7.5	7.5	7.5	7.5	7.5
PVPK29–32	15.0	15.0	15.0	15.0	15.0	15.0	15.0
M.S.	2.0	2.0	2.0	2.0	2.0	2.0	2.0

**Table 2:** Cumulative dissolution of core tablets and release of coating tablets in 0.5% SDS and water.

Molar ratios of vin and citric acid	1 : 0	1 : 1/3	1 : 2/3	1 : 1	1 : 4/3	1 : 6/3	1 : 7/3
Cumulative dissolution %	4.23	11.96	14.55	30.60	55.56	66.42	91.02
Cumulative release %	19.76	30.13	51.50	54.35	71.57	84.76	92.41

**Table 3:** Coating formulations.

Components	Quantity
CA 398-3	4.5 g
PEG-6000	1.35 g
Acetone*	95.0 mL
Diethyl-o-phthalate	1.0 mL
Water*	5.0 mL

Total	100.0 mL

*Major part of the acetone had been removed during coating. And the residual solvent acetone and water at 40°C for 24 h could be removed after coating.

**Table 4:** The pharmacokinetic parameters (vinpocetine) in dogs after oral administration of 15 mg vinpocetine osmotic pump controlled release tablets and immediate release-tablets (average ± SD, *n* = 6).

Parameters	Immediate release tablets	Osmotic pump controlled release tablets
*T*_max_ (h)	1.54 ± 0.64	4.83 ± 2.32*
*C*_max_ (ng/mL)	10.21 ± 1.55	3.62 ± 1.34*
AUC (ng/mL·min)	34.80 ± 22.52	40.87 ± 17.75**
*T*_1/2_ (h)	3.98 ± 1.06	3.77 ± 1.02**
MRT (h)	13.76 ± 1.5	26.27 ± 2.1*
Ke (h^−1^)	0.0756 ± 0.0164	0.0798 ± 0.0159**

*P < .05, compared to the immediate release tablets (*t*-test)

**P > .05, compared to the immediate release tablets (*t*-test).

**Table 5:** The pharmacokinetic parameters (vinpocetine metabolite apovincaminic acid) in dogs after oral administration of 15 mg vinpocetine osmotic pump controlled release tablets and immediate release-tablets (average ± SD, *n* = 6).

Parameters	Immediate release tablets	Osmotic pump controlled release tablets
*T*_max_ (h)	1.67 ± 0.31	4.83 ± 1.02*
*C*_max_ (ng/mL)	149.28 ± 31.22	52.01 ± 22.39*
AUC (ng/mL·min)	453.20 ± 57.55	377.19 ± 39.89**
T_1/2_ (h)	4.63 ± 0.64	4.17 ± 0.22**
MRT (h)	6.8 ± 0.86	17.29 ± 1.02*
Ke (h^−1^)	0.0851 ± 0.0493	0.1067 ± 0.0981**

*P < .05, compared to the immediate release tablets (*t*-test)

**P > .05, compared to the immediate release tablets (*t*-test).

## References

[1] Subhan Z, Hindmarch I (1985). Psychopharmacological effects of vinpocetine in normal healthy volunteers. *European Journal of Clinical Pharmacology*.

[2] Bönöczk P, Panczel G, Nagy Z (2002). Vinpocetine increases cerebral blood flow and oxygenation in stroke patients: a near infrared spectroscopy and transcranial Doppler study. *European Journal of Ultrasound*.

[3] Feigin VL, Doronin BM, Popova TF, Gribatcheva EV, Tchervov DV (2001). Vinpocetine treatment in acute ischaemic stroke: a pilot single-blind randomized clinical trial. *European Journal of Neurology*.

[4] McDaniel MA, Maier S, Einstein GO (2002). “Brainspecific” nutrients: a memory cure?. *International Psychological Science*.

[5] Szatmari SZ, Whitehouse PJ (2003). Vinpocetine for cognitive impairment and dementia. *Cochrane Database of Systematic Reviews*.

[6] Langer R (1998). Drug delivery and targeting. *Nature*.

[7] Ribeiro L, Ferreira DC, Veiga FJB (2005). In vitro controlled release of vinpocetine-cyclodextrin-tartaric acid multicomponent complexes from HPMC swellable tablets. *Journal of Controlled Release*.

[8] Ribeiro L, Veiga F (2002). Complexation of vinpocetine with cyclodextrins in the presence or absence of polymers. Binary and ternary complexes preparation and characterization. *Journal of Inclusion Phenomena and Macrocyclic Chemistry*.

[9] Nie S, Pan W, Li X, Wu X (2004). The effect of citric acid added to hydroxypropyl methylcellulose (HPMC) matrix tablets on the release profile of vinpocetine. *Drug Development and Industrial Pharmacy*.

[10] Cao GL, Wang J, Liu Y (2008). *In vivo* and *in vitro* evaluation of push-pull osmotic pump-controlled release tablet of vinpocetine using numerical deconvolution technique. *Chinese Journal of New Drugs*.

[11] Rose S, Nelson JF (1955). A continuous long-term injector. *The Australian Journal of Experimental Biology and Medical Science*.

[12] Theeuwes F (1975). Elementary osmotic pump. *Journal of Pharmaceutical Sciences*.

[13] Ayer A, Theeuwes F, Wong PS Process for increasing solubility of drug.

[14] Liu L, Wang X (2008). Solubility-modulated monolithic osmotic pump tablet for atenolol delivery. *European Journal of Pharmaceutics and Biopharmaceutics*.

[15] Gulyás B, Halldin C, Sóvágó J (2002). Drug distribution in man: a positron emission tomography study after oral administration of the labelled neuroprotective drug vinpocetine. *European Journal of Nuclear Medicine*.

